# Analysis of anticholinergic adverse effects using two large databases: The US Food and Drug Administration Adverse Event Reporting System database and the Japanese Adverse Drug Event Report database

**DOI:** 10.1371/journal.pone.0260980

**Published:** 2021-12-02

**Authors:** Junko Nagai, Yoichi Ishikawa

**Affiliations:** 1 The Office of Institutional Research, Meiji Pharmaceutical University, Kiyose, Tokyo, Japan; 2 Division of Clinical Pharmacy, Department of Pediatric Pharmaceutical Sciences, Education and Research Center for Pharmacy, Meiji Pharmaceutical University, Kiyose, Tokyo, Japan; National Chiao Tung University College of Biological Science and Technology, TAIWAN

## Abstract

**Introduction:**

Anticholinergic adverse effects (AEs) are a problem for elderly people. This study aimed to answer the following questions. First, is an analysis of anticholinergic AEs using spontaneous adverse drug event databases possible? Second, what is the main drug suspected of inducing anticholinergic AEs in the databases? Third, do database differences yield different results?

**Methods:**

We used two databases: the US Food and Drug Administration Adverse Event Reporting System database (FAERS) and the Japanese Adverse Drug Event Report database (JADER) recorded from 2004 to 2020. We defined three types of anticholinergic AEs: central nervous system (CNS) AEs, peripheral nervous system (PNS) AEs, and a combination of these AEs. We counted the number of cases and evaluated the ratio of drug–anticholinergic AE pairs between FAERS and JADER. We computed reporting odds ratios (RORs) and assessed the drugs using Beers Criteria^®^.

**Results:**

Constipation was the most reported AE in FAERS. The ratio of drug–anticholinergic AE pairs was statistically significantly larger in FAERS than JADER. Overactive bladder agents were suspected drugs common to both databases. Other drugs differed between the two databases. CNS AEs were associated with antidementia drugs in FAERS and opioids in JADER. In the assessment using Beers Criteria^®^, signals were detected for almost all drugs. Between the two databases, a significantly higher positive correlation was observed for PNS AEs (correlation coefficient 0.85, *P* = 0.0001). The ROR was significantly greater in JADER.

**Conclusions:**

There are many methods to investigate AEs. This study shows that the analysis of anticholinergic AEs using spontaneous adverse drug event databases is possible. From this analysis, various suspected drugs were detected. In particular, FAERS had many cases. The differences in the results between the two databases may reflect differences in the reporting countries. Further study of the relationship between drugs and CNS AEs should be conducted.

## Introduction

In recent years, as the proportion of the population of older adults has been increasing in developed countries, concern regarding pharmacotherapy for the elderly has been growing. The reason for this is elderly people tend to have many drug-related problems, including age-related changes in pharmacokinetics and pharmacodynamics and the concomitant use of multiple drugs [1]. These problems are considered to frequently cause drug adverse events in elderly people [2].

Anticholinergic adverse effects (AEs) in the elderly have been noted in many reports. Anticholinergic AEs are common drug adverse events in older adults over 65 years [3–5] and are classified as either peripheral side effects (e.g., constipation and dry mouth) or central side effects (e.g., delirium and cognitive dysfunction) [6]. It has been reported that around half of the older population uses at least one anticholinergic drug [7]. Drugs frequently prescribed for older adults for sleep disorders, anxiety, and urinary disorders often have antimuscarinic effects. The concomitant use of these drugs is thought to increase the risk of anticholinergic AEs due to their overlapping antimuscarinic effects [8]. Using anticholinergics in elderly persons leads to not only reductions in the patients’ quality of life but also an increased incidence of serious negative outcomes: falls, hospitalization, and an increased mortality rate [7,9–12]. For the above reasons, anticholinergic AE is an important problem in the pharmacotherapy of older adults.

Many researchers throughout the world have been conducting various studies on these issues [13,14]. In particular, research on anticholinergic burden scales is well known, and various kinds of anticholinergic burden scales have been developed and reported by many researchers. For instance, the ABC (Anticholinergic Burden Classification) [15], ACB (Anticholinergic Cognitive Burden scale) [16], and ARS (Anticholinergic Risk Scale) [17] are the major examples of anticholinergic burden scales. However, several problems with these scales have been pointed out: the strength of the anticholinergic effect is different for each scale, and not all of the drugs used around the world are listed in these scales [13,14].

Anticholinergic effects of all drugs should be assessed if possible. The reason for this is that drugs that are not categorized as antimuscarinic agents could induce anticholinergic AEs. Nowadays, in the field of drug development, much research on drug repositioning has been performed, and several papers have suggested that existing drugs have previously unsuspected pharmacological effects [18,19]. To examine this possibility, suspected drugs and cases of anticholinergic AEs should be explored widely. Many clinical studies have been conducted and published on the relationships between patients’ anticholinergic burden and the occurrence of anticholinergic AEs [12,20,21]. Nevertheless, the results have not always been consistent. The relationship between the anticholinergic burden scales and anticholinergic AEs is still unclear, and the validity of the anticholinergic burden scales remains unknown. Thus, several unclear points regarding anticholinergic AEs and their evaluation exist. Thus, studies of anticholinergic AEs using a large number of cases is necessary.

Recently, as high-performance computers have become widespread and data science has developed, big data research has been growing. Research with large clinical data in the clinical field, such as the analysis of spontaneous adverse drug event report databases, has also been increasing. Since 1992 when the term “Adverse Drug Reaction Reporting Systems” was introduced to MeSH, the number of articles including the term has increased [22]. The advantage of using a spontaneous adverse drug event report database is that researchers can gain abundant adverse drug event cases that occur in post-marketing clinical settings. These cases cannot be collected during premarketing clinical trials, as they emerge in the real world (i.e., patients with various comorbidities and concomitant drug use). In addition, these databases include a large number of adverse drug event cases that are difficult to collect from one or several centers as usual.

The aim of this study was to analyze anticholinergic AEs using large spontaneous adverse drug event report databases. We first investigated the number of cases of anticholinergic AEs to determine if these AEs could be analyzed using these databases, as AEs are not generally considered severe and cases that are not severe are seldom reported to spontaneous adverse drug event report databases. Second, we performed an analysis to reveal the major drugs suspected of causing anticholinergic AEs in current clinical settings. We used the Reporting Odds Ratio (ROR) method [23] to evaluate drugs that may cause anticholinergic AEs. It is one of the methods of disproportional analysis [24] that uses signal detection by regulatory agencies to detect early unknown adverse drug events, and it exhibits the highest sensitivity. Third, we evaluated the value of ROR using anticholinergic drugs that appeared in the AGS Beers Criteria^®^ [25] to assess the appropriateness of ROR for anticholinergic effects.

In this study, we used two spontaneous adverse drug event report databases: the US Food and Drug Administration Adverse Event Reporting System database (FAERS) [26] and the Japanese Adverse Drug Event Report database (JADER) [27]. FAERS was constructed by the Food and Drug Administration (FDA) in the US by collecting adverse drug event cases from around the world. JADER was constructed by the Pharmaceuticals and Medical Devices Agency (PMDA), the Japanese regulatory agency, by collecting adverse drug event cases in Japan since April 2004. These two databases are reported to have different features [28]. It is expected that their results will also be different owing to the different characteristics of the databases. Therefore, we analyzed the two databases separately and compared the results. FAERS has many non-serious AEs reported from non-health care professionals, while JADER has many serious AEs reported from medical professionals. FAERS includes various types of AEs regardless of the mechanism of action and JADER has major AEs that could be assumed from the mechanism. This suggests that we might be able to gain information about AEs due to unknown mechanisms from FAERS and information about serious AEs that are known inducing mechanisms from JADER. In addition, though AE reports in FAERS are registered from around the world, reports in JADER are limited to those in Japan; thus this difference may also lead to a different tendency of these databases. The usage of medicines also differs between Japan and the rest of the world; for instance, opioids are strictly regulated and benzodiazepines are frequently prescribed for many patients. Taking these factors into account, we compared the two databases.

## Materials and methods

### Databases

We used two large spontaneous adverse drug event report databases: FAERS and JADER. These databases are open to the public and freely available from each website after confirming the terms of use. The FAERS and JADER databases were downloaded, and they included reports recorded from January 2004 to December 2020 (2004Q1–2020Q4) and from April 2004 to December 2020, respectively. Both databases are anonymized; personal information was deleted from the reports and the cases were given an identification (ID) number to distinguish each individual when recorded in the database. Accordingly, a database user cannot identify a person recorded in the database.

### Definitions of anticholinergic AEs

AEs are recorded using the MedDRA [29] preferred term (PT) in each database. In this study, anticholinergic AEs were defined using the following nine PTs: cognitive disorder, confusional state, delirium, constipation, dry eye, dry mouth, thirst, tachycardia, and urinary retention. Then, these nine PTs were divided into three groups: the nine PTs were grouped as ALL, three central AE PTs (cognitive disorder, confusional state, and delirium) were grouped as central nervous system (CNS), and the other six peripheral AEs were grouped as peripheral nervous system (PNS). We analyzed these three groups of anticholinergic AEs.

### Construction of tables for analysis

Both FAERS and JADER are relational databases that are constructed with several data tables [30,31]. We used data tables that included drug names and AE names for analysis. We first performed data cleaning of the drug names in each database. Drug names recorded in FAERS were treated with data of Drugs@FDA [32] and Drugbank [33,34] to convert trade names to generic names. If a drug contained several active components, the data were divided into other data. Drug names in JADER recorded by only numeric characters or symbols were deleted. Next, we extracted the suspected drugs. A drug’s reported role in an event is recorded with the drugs in FAERS and JADER. The role types are “primary suspect drug,” “secondary suspect drug,” “concomitant,” and “interacting” in FAERS and “suspect drug,” “concomitant,” and “interacting” in JADER. In this analysis, we used primary suspect drug and secondary suspect drug in FAERS and suspect drug in JADER. After these treatments, two data tables that recorded drugs and adverse reactions were merged by each ID. Then, duplicated data were deleted following the procedure described in a previous report [35,36]. According to the above procedures, data tables for the analysis of both FAERS and JADER were constructed.

### Comparison of the number of cases between the two databases

We compared the number of cases of anticholinergic AEs recorded in FAERS and JADER to determine the differences. Each line in the data table for analysis constructed by the above procedure represented a drug–AE pair. We counted both the total number of lines (i.e., all drug–AE pairs) and the number of lines (drug–anticholinergic AE pairs) that included any PTs corresponding to the three AEs (ALL, CNS, PNS) in these data tables. We separately evaluated the ratio of the presence or absence of drug–anticholinergic AE pairs between FAERS and JADER according to the chi-square test of independence. We calculated the proportion of cases of anticholinergic AEs by the number of pairs in each data table to grasp the number of anticholinergic AE reports recorded in each database.

### Data analysis

**Fig 1:**
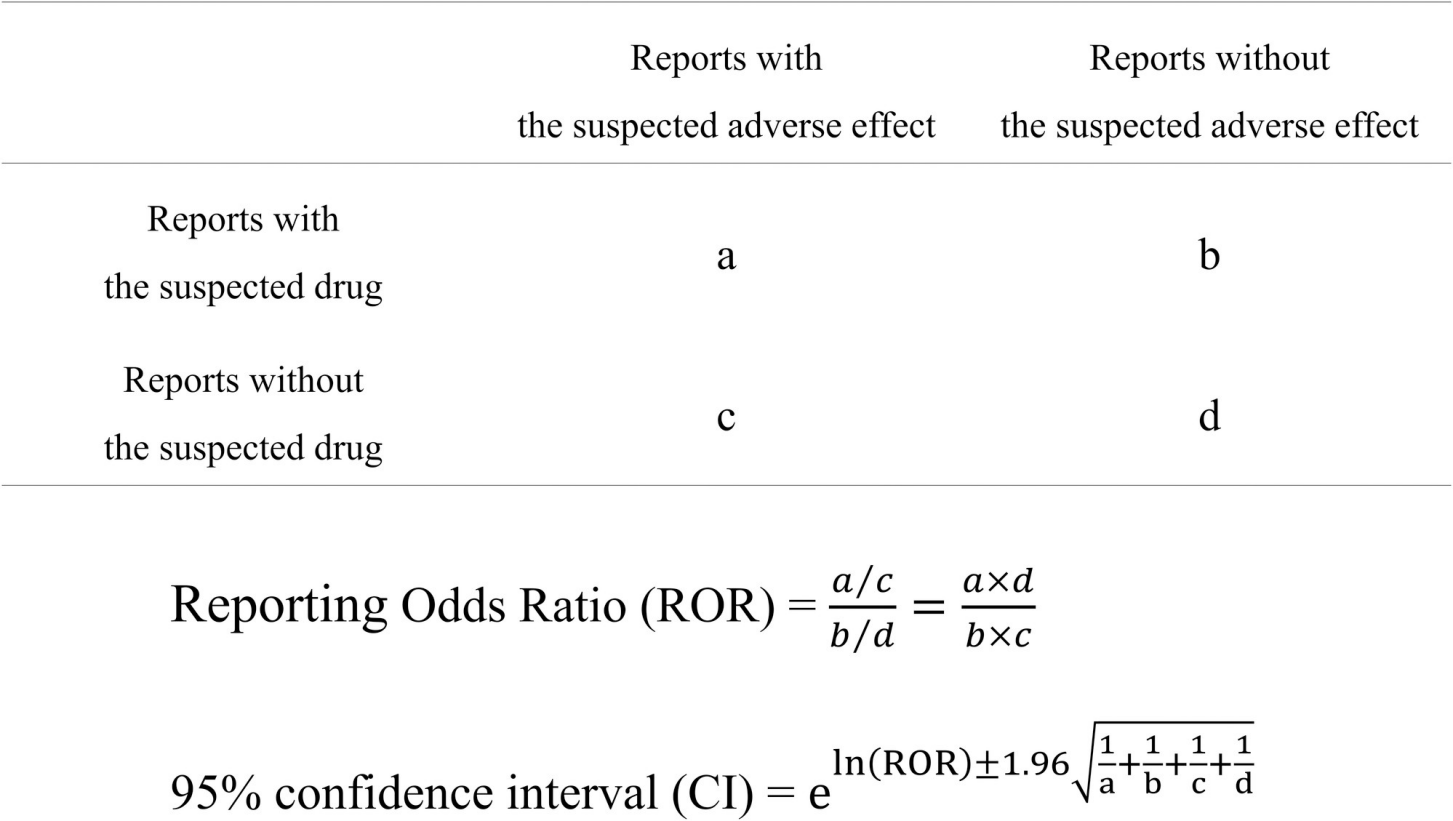
Two–by–two contingency table and calculation formula of ROR. The two–by–two contingency table contains reports with the suspected drug, reports without the suspected drug, reports with the suspected AE, and reports without the suspected AE (a–d indicate numbers of reports). The ROR and 95% CI were calculated as shown.

We calculated the RORs and 95% confidence intervals (CIs) from two-by-two contingency tables (Fig 1) and performed Fisher’s exact tests [37]. These were performed comprehensively for all combinations of drugs and three AEs in each data table by following previously reported procedures [36]. If a two-by-two contingency table contained zero in a cell, the ROR was not calculable, and the estimate was considered to be unstable if the number was too small. Therefore, we performed Haldane-Anscombe 1/2 corrections (i.e., the addition of 0.5 to all cells) to correct for this bias [38].

We plotted a volcano plot [39], a scatterplot using the natural logarithms of the RORs (lnROR) and the common logarithm of inverse *P*-values (−log *P*), to visualize the major suspected drugs.

The RORs, 95% CIs, and *P*-values of Fisher’s exact tests were summarized as results tables for both FAERS and JADER. We then extracted the major suspected drugs using the conditions shown below. The drugs whose lower limit of the 95% CI of the ROR was greater than 1 and the proportion of pairs was greater than 0.01% were extracted and sorted by *P-*value in ascending order. If the *P-*values of the drugs were the same, the drugs were sorted by ROR in descending order.

### Assessment based on list of Beers Criteria^®^

We evaluated the ROR values using drugs known to possess anticholinergic effects. These anticholinergics were adopted from “Drugs with Strong Anticholinergic Properties” in AGS Beers Criteria^®^ updated by the American Geriatrics Society in 2019 [25]. We only evaluated the drugs for which the ROR could be calculated for both FAERS and JADER. Clidinium-chlordiazepoxide was separated into clidinium and chlordiazepoxide and they were treated independently.

We first extracted drugs whose *P*-value obtained through Fisher’s exact test was less than 0.05 from each results table. The drugs were separated into two groups: anticholinergics of Beers Criteria^®^ (Group B) and other drugs (Group non-B). We plotted box plots and observed differences in the distribution of lnROR among groups by each AE. These are the outlier box plots. The whiskers extend from the ends of the box to the outermost data point that falls within the distances computed as follows: 1st quartile—1.5× (interquartile range), 3rd quartile + 1.5× (interquartile range). If the data points do not reach the computed ranges, then the whiskers are determined by the upper and lower data point values (not including outliers) [40]. The median lnRORs were compared between the two groups, and the Wilcoxon rank sum test was performed. Second, we computed Spearman’s correlation coefficient to examine the lnROR trends of the Group B drugs between the two databases by the three anticholinergic AEs. Third, we assessed the differences in the lnROR between the two databases by all three AEs using the Wilcoxon signed-rank test.

### Statistical analysis

All data analyses were performed using JMP^®^Pro15.2.1 (SAS Institute Inc., Cary, NC, USA). Any *P*-value <0.05 was considered significant.

## Results

### Number of cases

The number of all drug–AE pairs was 102,916,650 in FAERS and 1,807,801 in JADER. The numbers of pairs are shown in Table 1.

**Table 1:** Numbers of drug–AE pairs.

Number of pairs	FAERS		JADER		
	102,916,650		1,807,801		
Adverse effects	N^a)^	(%) ^b)^	N^a)^	(%) ^b)^	*P*- value^c)^
**ALL**	1,110,103	(1.08)	15,605	(0.86)	< 0.0001
**CNS**	391,053	(0.38)	6,175	(0.34)	< 0.0001
**Cognitive disorder**	81,707	(0.08)	673	(0.04)	< 0.0001
**Confusional state**	255,630	(0.25)	647	(0.04)	< 0.0001
**Delirium**	53,716	(0.05)	4,855	(0.27)	< 0.0001
**PNS**	719,050	(0.70)	9,430	(0.52)	< 0.0001
**Constipation**	329,600	(0.32)	1,858	(0.10)	< 0.0001
**Dry eye**	57,090	(0.06)	91	(0.01)	< 0.0001
**Dry mouth**	118,195	(0.11)	111	(0.01)	< 0.0001
**Tachycardia**	138,333	(0.13)	1,927	(0.11)	< 0.0001
**Thirst**	23,440	(0.02)	340	(0.02)	0.0004
**Urinary retention**	52,392	(0.05)	5,103	(0.28)	< 0.0001

^a)^ Number of pairs.

^b)^ Proportion of cases of anticholinergic AEs by the number of pairs. ^c)^ *P*–value of the chi–square test.

The ratio of drug–anticholinergic AE pairs that had statistically significantly differences between FAERS and JADER were recorded. Delirium and urinary retention were more common in JADER than in FAERS, while other AEs were more common in FAERS than in JADER. Constipation was the most reported AE in FAERS (the proportion of pairs was 0.32%), followed by a confusional state (0.25%), and thirst was the least common (0.02%). Urinary retention was the most common AE in JADER (0.28%), and the least common was dry eye (0.01%).

### Major suspected drugs

**Fig 2:**
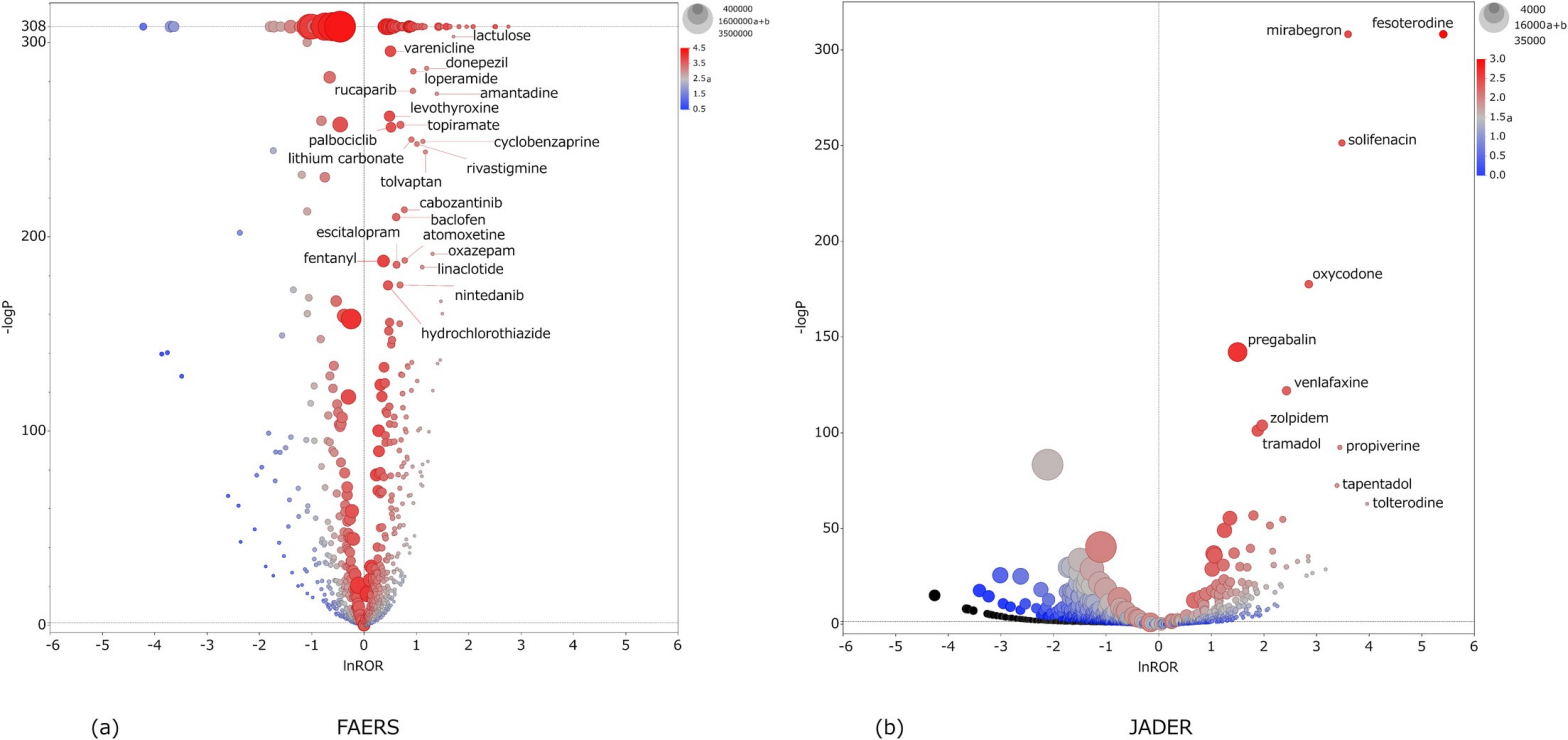
Scatterplot of ALL. Tendency of inducing all anticholinergic AEs (ALL) by each drug. (a) FAERS and (b) JADER.

**Fig 3:**
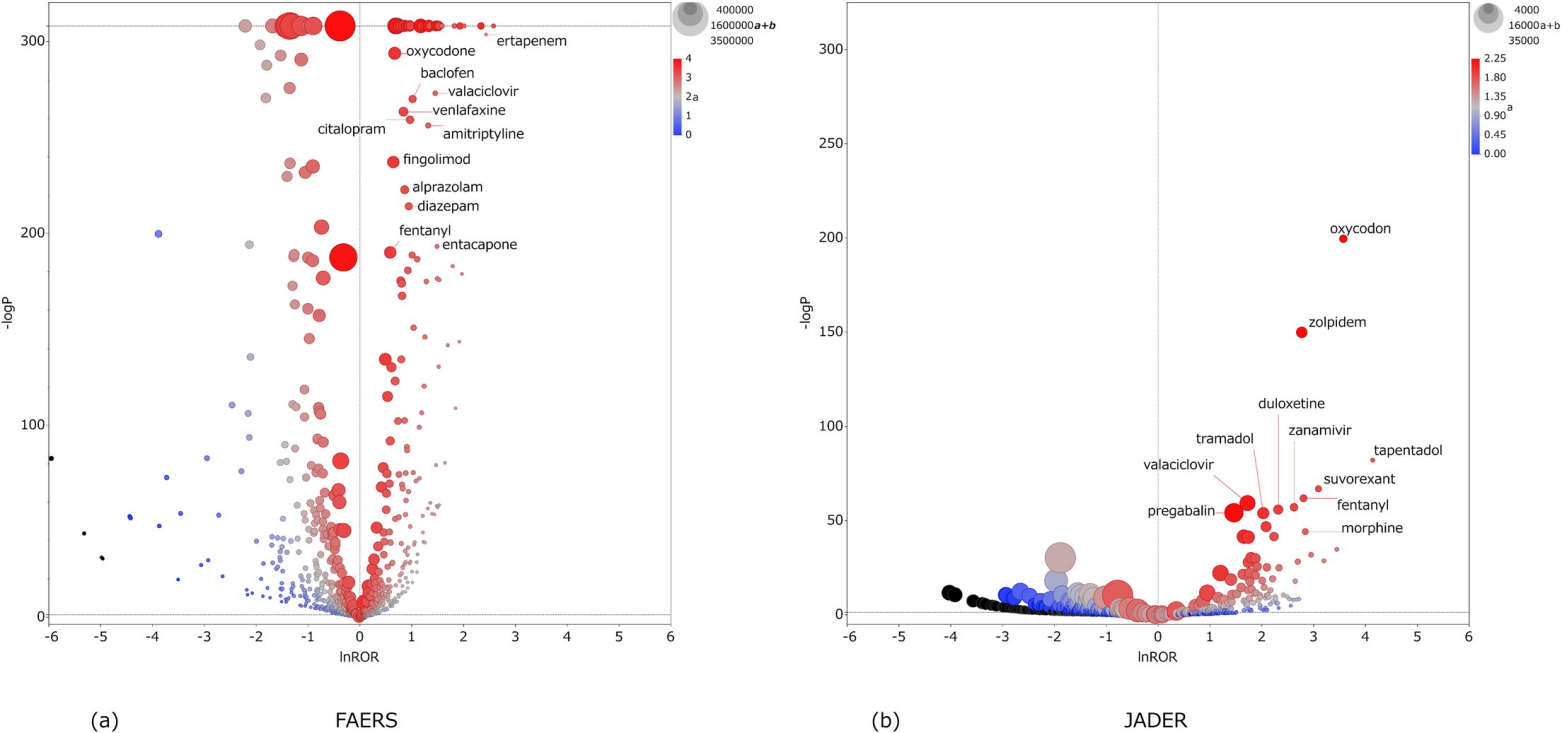
Scatterplot of CNS. Tendency of inducing CNS anticholinergic AEs by each drug. (a) FAERS and (b) JADER.

**Fig 4:**
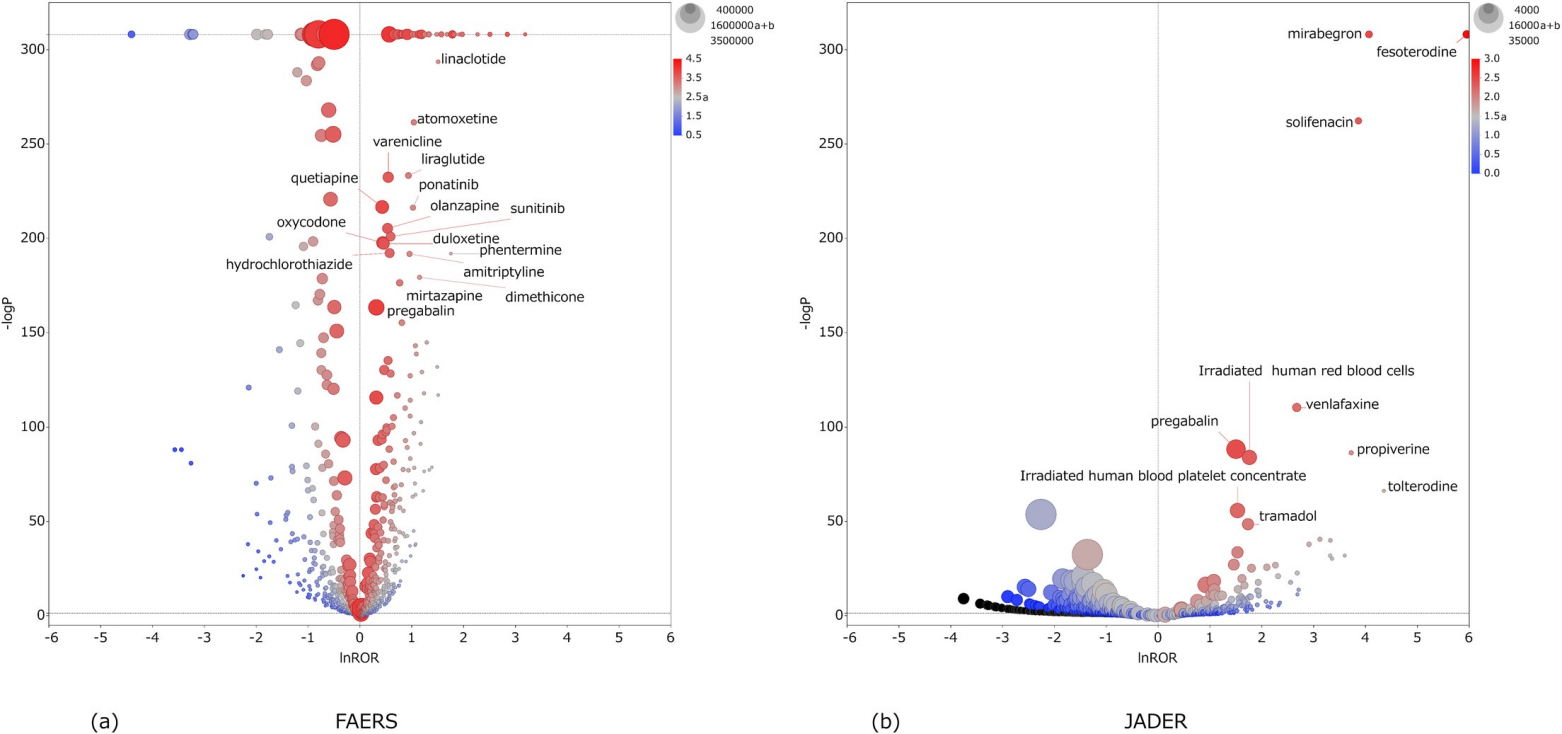
Scatterplot of PNS. Tendency of inducing PNS anticholinergic AEs by each drug. (a) FAERS and (b) JADER.

Figs 2–4 are scatterplots plotted by lnROR and −log *P*. Each dot is a drug, and the abscissa of the scatterplot is the lnROR axis. The ordinate of the scatterplot is the −log *P* axis, and the *P*-value of 0.05 is represented by the dotted line of the ordinate.

As lnROR becomes more positive, there is a greater tendency of anticholinergic AEs, and the increasing −log *P* indicates that these are more statistically significant. The size of the dots reflects the number of cases of each drug: it is the value of “a+b” in the two-by-two contingency table. The color of the dots indicates the number of cases of anticholinergic AEs of the drug: it is the value of “a” in the two-by-two table. Accordingly, drugs plotted in the top-right of the scatterplot and in red are the major suspected drugs. Tables 2–4 show the major suspected drugs.

**Table 2:** Major suspected drugs of ALL. (a) FAERS (b) JADER.

(a) FAERS							
	**drug**	**a^a)^**	**(a/(a+c^b)^)) ×100**	**ROR^c)^**	**95% CI**	***P*-value^d)^**	**−log*P*^e)^**
**1**	sevelamer	2,727	0.25	15.9	(15.2–16.5)	< 0.0001	308
**2**	solifenacin	4,913	0.44	12.3	(12–12.7)	< 0.0001	308
**3**	fesoterodine	2,713	0.24	12.1	(11.7–12.6)	< 0.0001	308
**4**	patiromer	3,197	0.29	8.1	(7.8–8.4)	< 0.0001	308
**5**	tolterodine	1,778	0.16	7.1	(6.8–7.5)	< 0.0001	308
**6**	memantine	2,332	0.21	6.2	(5.9–6.4)	< 0.0001	308
**7**	mirabegron	2,736	0.25	5.2	(5–5.4)	< 0.0001	308
**8**	pimavanserin	6,080	0.55	4.8	(4.7–5)	< 0.0001	308
**9**	telotristat ethyl	1,030	0.09	4.5	(4.2–4.8)	< 0.0001	308
**10**	oxybutynin	1,411	0.13	4.5	(4.2–4.7)	< 0.0001	308
(b) JADER							
	**drug**	**a** ^**a)**^	**(a/(a+c** ^**b)**^ **)) ×100**	**ROR** ^**c)**^	**95% CI**	***P*-value** ^**d)**^	−**log*P***^**e)**^
**1**	fesoterodine	1,098	7.04	224.0	(202.6–247.7)	< 0.0001	308
**2**	mirabegron	315	2.02	36.6	(32.3–41.6)	< 0.0001	308
**3**	solifenacin	241	1.54	32.6	(28.2–37.6)	< 0.0001	251
**4**	oxycodone	220	1.41	17.4	(15.1–20.0)	< 0.0001	178
**5**	pregabalin	459	2.94	4.5	(4.1–4.9)	< 0.0001	142
**6**	venlafaxine	188	1.2	11.4	(9.8–13.2)	< 0.0001	122
**7**	zolpidem	218	1.4	7.2	(6.3–8.2)	< 0.0001	104
**8**	tramadol	227	1.45	6.6	(5.8–7.5)	< 0.0001	101
**9**	propiverine	89	0.57	31.4	(24.8–39.6)	< 0.0001	92
**10**	tapentadol	71	0.45	29.7	(22.9–38.6)	< 0.0001	72

These drugs ranked in the top 10 in the ALL result. (a) FAERS and (b) JADER.

^a)^ It is the “a” in the two–by–two table (i.e., number of cases reporting the suspected AE and the suspected drug).

^b)^ It is the “c” in the two–by–two table (i.e., number of cases reporting with the suspected AE and without the suspected drug).

^c)^ Reporting odds ratio.

^d)^ *P*–value of Fisher’s exact test.

^e)^ Common logarithm of the inverse *P*–values. If it was not indicated, it was substituted with 308.

**Table 3:** Major suspected drugs of CNS. (a) FAERS (b) JADER.

(a) FAERS							
	**drug**	**a^a)^**	**(a/(a+c^b)^)) ×100**	**ROR^c)^**	**95% CI**	***P*-value^d)^**	**−log*P*^e)^**
**1**	memantine	1,776	0.45	13.2	(12.6–13.9)	< 0.0001	308
**2**	pimavanserin	4,587	1.17	10.4	(10.1–10.7)	< 0.0001	308
**3**	donepezil	1,055	0.27	7.5	(7–8)	< 0.0001	308
**4**	finasteride	3,078	0.79	6.9	(6.7–7.2)	< 0.0001	308
**5**	rivastigmine	1,206	0.31	6.2	(5.9–6.6)	< 0.0001	308
**6**	oseltamivir	913	0.23	4.9	(4.6–5.2)	< 0.0001	308
**7**	lorazepam	2,120	0.54	4.6	(4.4–4.8)	< 0.0001	308
**8**	zolpidem	2,000	0.51	4.5	(4.3–4.7)	< 0.0001	308
**9**	carbidopa	5,257	1.34	4.5	(4.4–4.7)	< 0.0001	308
**10**	lithium carbonate	1,186	0.30	4.5	(4.2–4.8)	< 0.0001	308
(b) JADER							
	**drug**	**a** ^**a)**^	**(a/(a+c** ^**b)**^ **)) ×100**	**ROR** ^**c)**^	**95% CI**	***P*-value** ^**d)**^	−**log*P***^**e)**^
**1**	oxycodone	180	2.91	35.7	(30.6–41.8)	< 0.0001	200
**2**	zolpidem	190	3.08	16.0	(13.8–18.6)	< 0.0001	150
**3**	tapentadol	61	0.99	63.0	(47.8–83.1)	< 0.0001	82
**4**	suvorexant	72	1.17	22.1	(17.4–28.1)	< 0.0001	67
**5**	fentanyl	76	1.23	16.6	(13.2–20.9)	< 0.0001	62
**6**	valacyclovir	150	2.43	5.6	(4.8–6.6)	< 0.0001	59
**7**	zanamivir	77	1.25	13.8	(11.0–17.4)	< 0.0001	57
**8**	duloxetine	90	1.46	10.2	(8.2–12.6)	< 0.0001	56
**9**	pregabalin	178	2.88	4.3	(3.7–5.0)	< 0.0001	54
**10**	tramadol	106	1.72	7.6	(6.3–9.3)	< 0.0001	54

These drugs ranked in the top 10 in the CNS result. (a) FAERS and (b) JADER.

^a)^ It is the “a” in the two–by–two table (i.e., number of cases reporting the suspected AE and suspected drug).

^b)^ It is the “c” in the two–by–two table (i.e., number of cases reporting the suspected AE and without the suspected drug).

^c)^ Reporting odds ratio.

^d)^ *P*–value of Fisher’s exact test.

^e)^ Common logarithm of inverse *P*–values. If it was not indicated, it was substituted with 308.

**Table 4:** Major suspected drugs of PNS. (a) FAERS (b) JADER.

(a) FAERS							
	**drug**	**a^a)^**	**(a/(a+c^b)^)) ×100**	**ROR^c)^**	**95% CI**	***P*-value^d)^**	**−log*P*^e)^**
**1**	sevelamer	2,701	0.38	24.3	(23.4–25.3)	< 0.0001	308
**2**	solifenacin	4,473	0.62	17.2	(16.7–17.8)	< 0.0001	308
**3**	fesoterodine	2,500	0.35	17.2	(16.5–17.9)	< 0.0001	308
**4**	patiromer	3,149	0.44	12.3	(11.9–12.8)	< 0.0001	308
**5**	tolterodine	1,575	0.22	9.7	(9.2–10.2)	< 0.0001	308
**6**	mirabegron	2,475	0.34	7.2	(6.9–7.5)	< 0.0001	308
**7**	telotristat ethyl	1,001	0.14	6.8	(6.4–7.2)	< 0.0001	308
**8**	erenumab	3,142	0.44	6.1	(5.9–6.4)	< 0.0001	308
**9**	niraparib	6,704	0.93	6.0	(5.8–6.1)	< 0.0001	308
**10**	oxybutynin	1,108	0.15	5.4	(5.1–5.7)	< 0.0001	308
(b) JADER							
	**drug**	**a** ^**a)**^	**(a/(a+c** ^**b)**^ **)) ×100**	**ROR** ^**c)**^	**95% CI**	***P*-value** ^**d)**^	−**log*P***^**e)**^
**1**	fesoterodine	1,093	11.59	386.2	(349.0–427.4)	< 0.0001	308
**2**	mirabegron	303	3.21	58.6	(51.4–66.7)	< 0.0001	308
**3**	solifenacin	217	2.30	47.8	(41.2–55.5)	< 0.0001	262
**4**	venlafaxine	147	1.56	14.6	(12.3–17.2)	< 0.0001	110
**5**	pregabalin	281	2.98	4.5	(4.0–5.1)	< 0.0001	88
**6**	propiverine	74	0.78	41.6	(32.3–53.4)	< 0.0001	86
**7**	Irradiated human red blood cells	207	2.20	5.8	(5.1–6.7)	< 0.0001	84
**8**	tolterodine	47	0.50	78.1	(55.7–109.6)	< 0.0001	66
**9**	Irradiated human blood platelet concentrate	170	1.80	4.6	(4.0–5.4)	< 0.0001	56
**10**	tramadol	121	1.28	5.7	(4.7–6.8)	< 0.0001	48

These drugs ranked in the top 10 in the PNS result. (a) FAERS and (b) JADER.

^a)^ It is the “a” in the two–by–two table (i.e., number of cases reporting the suspected AE and with the suspected drug).

^b)^ It is the “c” in the two–by–two table (i.e., number of cases reporting the suspected AE and without the suspected drug).

^c)^ Reporting odds ratio.

^d)^ *P*–value of Fisher’s exact test.

^e)^ Common logarithm of inverse *P*–values. If it was not indicated, it was substituted with 308.

**Fig 5:**
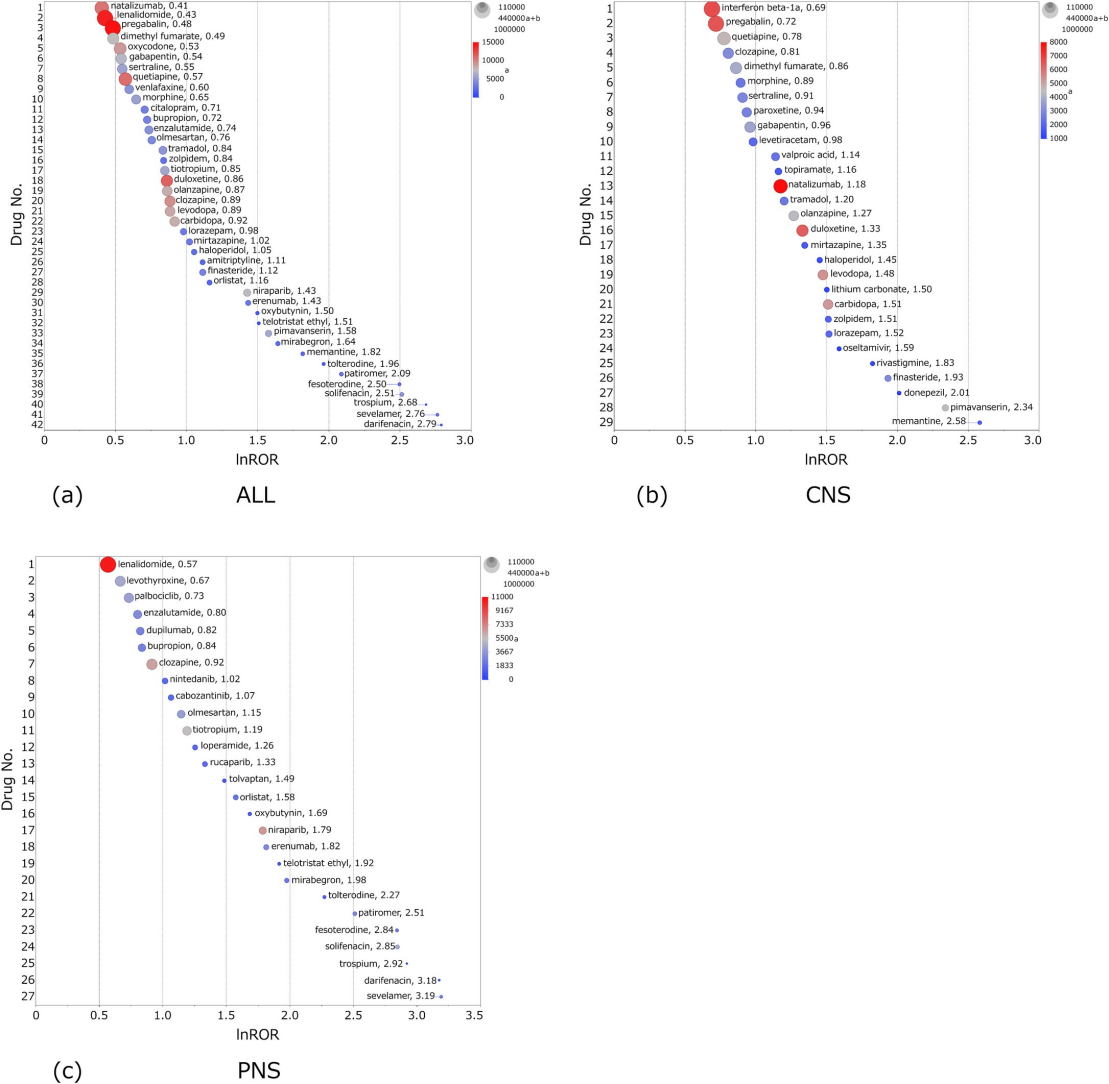
Plots of drugs whose −log *P* is 308. Drugs whose −log *P* is 308 and lnRORs are greater than 0 in FAERS: (a) ALL, (b) CNS, and (c) PNS. The size of the dots reflects the number of cases of each drug: It is “a+b” in the two–by–two contingency table. The color of the dots indicates the number of cases of anticholinergic AEs of the drug: It is “a” in the two–by–two table. Each dot is a drug, and the abscissa is the lnROR axis. The ordinate axis is the drug number by the descending lnROR.

Several major suspected drugs in each AE differed between FAERS and JADER. Several *P*-values of drugs were not able to be shown in FAERS because the values were extremely near to zero that could not be indicated on JMP. The smallest *P*-value that can be indicated on JMP is minus 2 to the power of 1022 and the common logarithm of inverse this value is 308. For these *P-*values, −log *P* was substituted as 308. Fig 5 illustrates the drugs whose −log *P* was 308 and the lnROR was greater than 0. These drugs are shown in the S1–S3 Tables.

According to Figs 2–4 and Tables 2–4, it can be seen that many drugs whose lnROR is greater than 0 and *P*-value is less than 0.05 are found in FAERS in comparison with only a few in JADER. The value of (a/(a+c)) ×100 for each drug tended to be smaller in FAERS than JADER. Many drugs had a positive lnROR in FAERS; however the range of lnROR was narrow and the maximum ln ROR was 3.19 for sevelamer in PNS. In JADER, the range of lnROR was wide and the maximum was 5.96 of fesoterodine in PNS. Fig 5 shows the drugs whose -log*P* is 308. The drugs which had a larger lnROR tended to be a small dot and a blue color. The small dot means that the drugs had a small number of cases regardless of the type of AEs and the blue color means that the drugs had a small proportion of anticholinergic AEs against the cases registered as a suspected drug, respectively. In the ALL results, three overactive bladder agents (solifenacin, fesoterodine, and mirabegron) were common to both FAERS and JADER. Other major suspected drugs of ALL were polymers in FAERS and opioids in JADER. Zolpidem was the common major suspected drug in CNS. The other remarkable drugs of CNS were dementia therapeutic agents in FAERS and analgesic drugs including opioids in JADER. In PNS, overactive bladder agents were common, as in ALL; others included polymers in FAERS and blood preparations in JADER. Tramadol and pregabalin were ranked in the top 10 of ALL, CNS, and PNS result in JADER. There were no drugs ranked in the top 10 of the three AEs in FAERS.

### Assessment based on the list of Beers Criteria^®^

**Fig 6:**
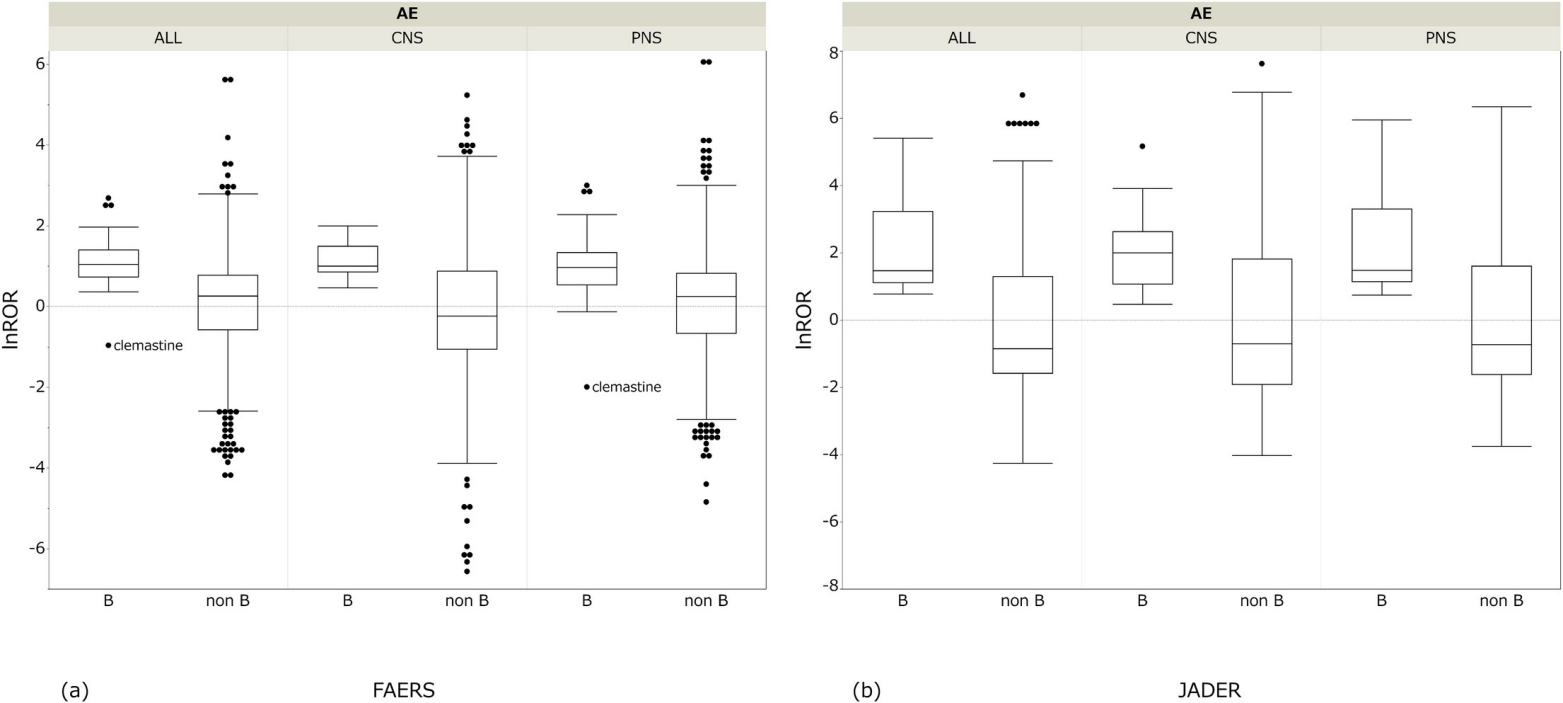
Comparison of Beers Criteria^®^ anticholinergic drugs and other drugs. These box plots show the distribution of the lnROR by the anticholinergic drugs and the other drugs. (a) FAERS and (b) JADER.

Fig 6 shows the results of the comparison of Beers Criteria^®^ anticholinergic agents by each AE in FAERS or JADER.

The median of Group B was greater than that of Group non-B, and the statistical significance of the difference was shown in all combinations of AEs and databases. All drugs of Group B had an lnROR greater than 0 in JADER; on the other hand, drugs whose lnROR was less than 0 existed in FAERS. The lnROR of ALL and PNS of clemastine and PNS of paroxetine showed negative values.

The results of the correlation of lnROR by AEs between FAERS and JADER were as follows. In PNS, a significantly high positive correlation 0.85 (*P* = 0.0001) was observed. There was a significantly positive correlation in ALL (0.63, *P* = 0.0028) and an insignificant weak positive correlation in CNS (0.23, *P=*0.41). In CNS, the median of lnROR was 1.03 (minimum 0.55-maximum 1.66) in FAERS and 2.11 (0.47–5.17) in JADER. The range of lnROR was wider in JADER than FAERS.

**Fig 7:**
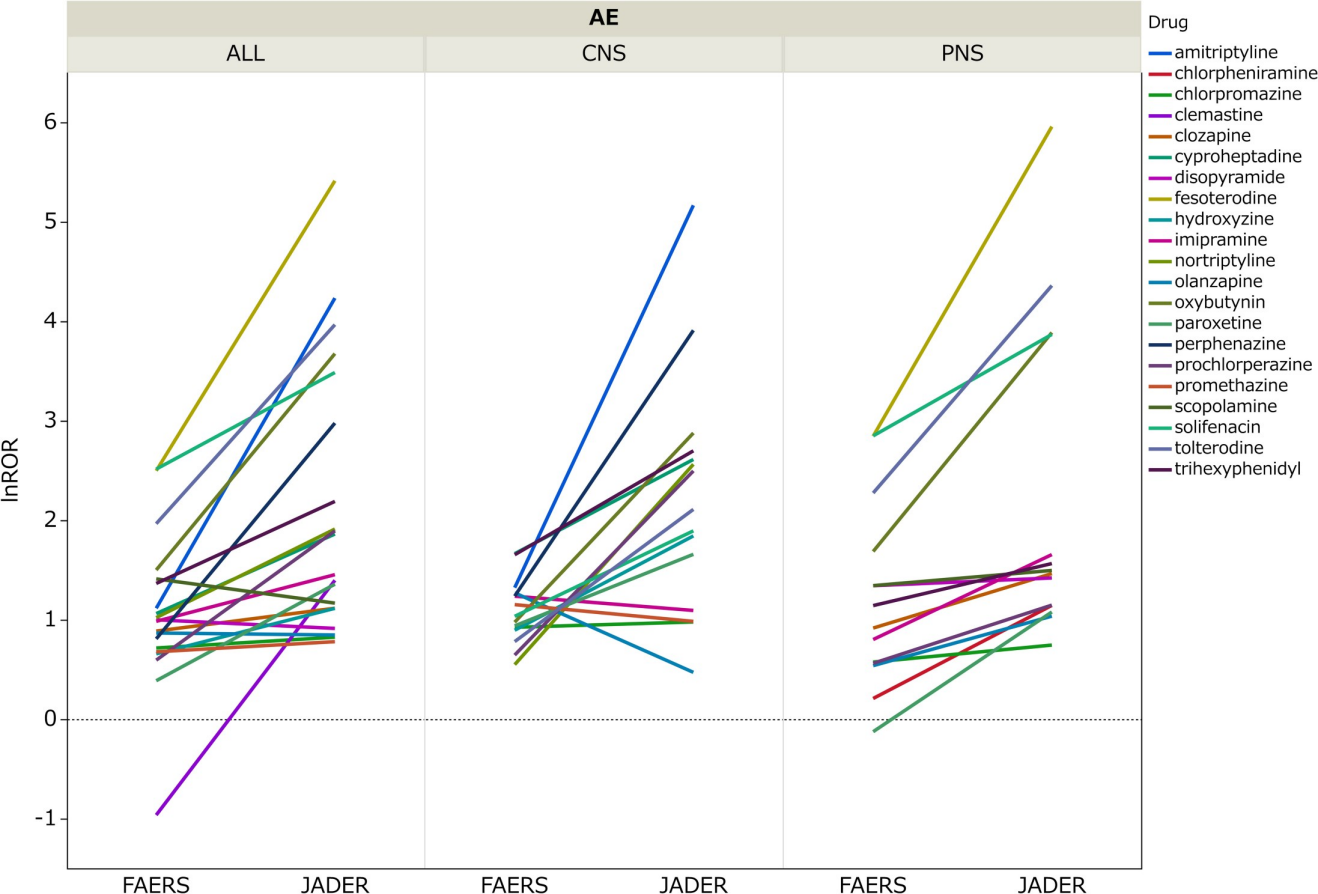
Comparison of the lnROR between FAERS and JADER. The lnROR of FAERS and JADER by each drug. Each line is a drug.

Fig 7 shows the difference in the lnROR means between the two databases by all three AEs using the Wilcoxon signed-rank test. As the drugs whose *P*-value was less than 0.05 were extracted and evaluated, a total of 21 drugs were shown. Most lnRORs of the three AEs were significantly greater in JADER. The greatest difference in terms of the absolute value was 3.84 for amitriptyline of CNS. The smallest was 0.02 for olanzapine of ALL.

## Discussion

### Number of cases

Based on the number of pairs and IDs, it became clear that FAERS and JADER included a larger number of cases of anticholinergic AEs than any single- or multi-center clinical studies. The analysis of a spontaneous adverse drug event reporting database would be applicable to research on anticholinergic AEs as other AEs because the ROR of anticholinergic AEs could be calculated. The proportion of anticholinergic AE cases in the whole database was not large, and it was especially low in JADER. This result is consistent with a previous report indicating that most cases recorded in JADER are severe AEs [41].

### Major suspected drugs

The drugs for the treatment of pollakiuria were common major suspected drugs of ALL and PNS. These drugs are particularly important for considering anticholinergic AEs in that they are mostly prescribed to elderly people. In a systematic review, Durán classified oxybutynin and tolterodine as high potency anticholinergics based on seven anticholinergic scores [15,17,42–46]. Solifenacin and fesoterodine were ranked in the major suspected drugs of our analysis; nevertheless, these drugs did not appear in Durán’s list. The anticholinergic scores used in Durán’s systematic review were published from 2006 to 2011 and the International Birth Date of solifenacin and fesoterodine were 2004 and 2007, respectively; therefore, it seems that when these anticholinergic scores were created, these new drugs were not thoroughly evaluated. As Lisibach pointed out [14], these major anticholinergic scores were developed in the early 2000s, and it is inferred that the trend of drug prescription and administration and the occurrence of AEs have been changing over time. Taking these changes into account, these anticholinergic scores might be not able to cover the currently major drugs inducing anticholinergic AEs. These scores need to be updated as is the case Beers Criteria. The results of our study suggest that the analysis using the spontaneous adverse drug event report database might be utilized for one of the assessment tools of clinical anticholinergic AEs. Other major suspected drugs differed between FAERS and JADER. The cause of this difference may be differences in the prescription of these drugs or the reporting of cases instead of the mechanism of the drugs. For instance, opioids are major suspected drugs of CNS in JADER. In Japan, the prescription and inventory management of opioids has always been under very strict control by law, and their indications are limited mostly to cancer pain and some types of chronic pain [47]. Consequently, regarding the AE reports of opioids in JADER, it is considered that the AEs induced by appropriate usage (constipation, delirium, etc.) are frequent and those induced by inappropriate use (addiction, abuse, etc.) are quite rare. Thus, the RORs of ALL and CNS are large. In contrast, the AE reports of opioids in FAERS are also obtained from various countries in which the regulation of opioids is less strict. Therefore, the RORs of anticholinergic AEs are smaller. Interestingly, tramadol and pregabalin were ranked in the top 10 of three AEs in JADER. These two drugs are prescription drugs for pain but are not specially regulated by law; therefore, they have been prescribed to many patients in recent years in Japan. The reason for these being drugs ranked in JADER is as follows. Both drugs have notes to pay attention to central nervous system AEs in the package insert. Tramadol has a high risk of inducing constipation because it is an opioid analgesic and pregabalin has been reported in cases of constipation and tachycardia. However, to clearly prove the connection more research is needed. These two drugs are likely to be prescribed to elderly persons because most of them have some pain so that we need to be aware of the inappropriate usage of these drugs.

The major suspected drugs in CNS were observed to have different trend types of medicines in ALL or PNS contrary to our expectations. Typical anticholinergics were not listed in the major suspected drugs of CNS; instead, cholinesterase inhibitors were ranked in FAERS, and antiviral drugs were ranked in JADER. However, the accumulation of anticholinergic effects might be one of the factors inducing central AEs, since several drugs with anticholinergic effects are included in the major suspected drugs [48]. The patient’s background factors may also need to be considered, as we adopted “cognitive impairment” and “confusional state” as CNS in this analysis. Several prior clinical studies have suggested no relationship between anticholinergic effects and cognitive disfunction [49,50]. Thus, these CNS symptoms might not be anticholinergic AEs. These previous studies and results of our study lead us to presume that revealing the relationship of AEs of CNS and drugs is a very difficult challenge. AEs of CNS are a serious problem not only for the patients, but also their families, and caregivers. In any case, the relationship between anticholinergic effects and CNS AEs should be studied further. It became clear from our study that spontaneous adverse drug event report databases include a large number of anticholinergic AEs; thus we would like to perform a continuous study that considered an accumulation of the drug burden and patient characteristics.

### Beers Criteria^®^ anticholinergic drugs

The fact that the lnRORs of almost all anticholinergics of Beers Criteria^®^ are greater than 0 indicates that these drugs induce anticholinergic AEs. This result indicates that the ROR helps assess drugs that have anticholinergic AEs. For this reason, the spontaneous adverse drug event report database can be applied for research on anticholinergic AEs.

The lnRORs of anticholinergics of the Beers Criteria^®^ tended to be greater in JADER than in FAERS. This difference of lnROR is considered to be the reason for the insignificant weak correlation in CNS. The values of the lnROR result of JADER in CNS were widely distributed in comparison with that of FAERS. Major drugs that had a large value of the lnROR results of JADER in CNS were amitriptyline, perphenazine, and oxybutynin. These drugs are listed in “List of drugs to be prescribed with special caution” in the “Guidelines for medical treatment and its safety in the elderly 2015” [51] published by The Japan Geriatrics Society. In this list, these drugs are flagged to be careful of cognitive impairment and delirium. It can be presumed that the cases of JADER are reported drugs that have antimuscarinic effects because they are frequently reported by healthcare professionals, and that leads to larger RORs. If the difference in the value of ROR reflects the actual status of drug use, it conforms to the opinion that the anticholinergic rating scale differs for each country [52]. It should be noted that this is only a hypothesis and further study to pursue the reason for the differences in the lnROR between the two databases and weak correlations in CNS should be conducted.

### Limitations

Our study has several limitations. First, a study using a spontaneous adverse drug event reporting database has various biases [53,54]. Clearly, only reported AE cases were recorded in the databases, while unreported cases could not be obtained from the databases. Almost the cases in the database were not verify for a causal association. Hence, it is not always said that the drugs caused the side effects. Second, these cases might have been affected by concomitant drug use and comorbidities. These factors can affect the incidence of AEs, although we analyzed only suspected drugs in this study. Mirabegron is a β3 agonist, and it led to the same number of anticholinergic AEs as a placebo according to a clinical study [55]. Its high ROR in this study may have been influenced by these factors.

Some drugs that are not classified as antimuscarinics might have the possibility to possess antimuscarinic mechanisms inducing anticholinergic AEs and it is necessary to verify these drugs using a chemical structure for the affinity of the muscarinic receptor. Moreover, the symptoms of anticholinergic AEs defined in this study are not always caused by antimuscarinic effects; that is, other pharmacological mechanisms can also cause them. However, the point is that the problems of giving prescriptions to the elderly in developed countries are symptoms with a phenotype of anticholinergic AEs. Whether antimuscarinic effects cause the symptoms is not the main point of argument. From this point of view, it is important to note that this study quantified the phenotype of anticholinergic AEs.

As we have indicated in the results section, the number of cases and the results of the analyses have commonalities and differences between the two databases. In the results of the major suspected drug, the value of (a/(a+c)) ×100 tended to be smaller in FAERS, which indicated that many suspected drugs were reported to have one AE. This suggests that various AE cases, which were not biased a specific suspected drug, were reported widely. In the results of JADER, limited several major suspected drugs had a large lnROR. This implies that cases of anticholinergic AEs in JADER were converged on some typical drugs. These results suggest that the databases must be chosen properly for the purpose of analysis. For example, it is suggested that FAERS might be suitable to detect an unknown mechanism of a drug because there are a large number of cases and the cases are not constrained by already known pharmacological actions, while JADER might be suitable to research the actual incidence of AEs and to evaluate AEs based on already known mechanisms. Needless to say, JADER will also apply to research local settings in Japan. Moreover, collating the results from these databases might help to elucidate AEs that happen in the real world.

Clearly, anticholinergic AEs are a problem that troubles patients and caregivers in developed countries, as numerous studies have reported to date. However, the assessment of the association between anticholinergic AEs and medications is generally difficult because almost all of these AEs lack objective parameters, like laboratory data or data from imaging studies, and similar symptoms result from physiological changes caused by aging. It seems that these facts can explain the variation in the many studies performed on anticholinergic AEs.

A further study of anticholinergic burden would be of value to the field of pharmacotherapy for the elderly. We hope that this study using spontaneous adverse drug event report databases will contribute to a solution to the problem. The idea of data mining is to find new knowledge without being hindered by the noise. In particular, the analysis of spontaneous adverse drug event reporting databases combined with other methods will result in more useful outcomes.

## Conclusions

A sufficient number of cases of anticholinergic AEs to perform a disproportional analysis was recorded in FAERS and JADER. The RORs of anticholinergics were assessed using Beers Criteria^®^. Using a spontaneous adverse drug event reporting database can be an effective method of anticholinergic AEs research in that it is an efficient way to collect a large number of cases. It is important to select databases depending on the aim of the research because the results of our analysis differed between the two databases. We recommend FAERS to search for a wide range of suspect drugs including unknown mechanisms of drugs and JADER to understand the real current status of known AEs or situations in Japan.

This study indicates that the principal suspected drugs of anticholinergic AEs are those used for the treatment of pollakiuria. The relationship of anticholinergic AEs of CNS and drugs should be studied further, including the patient’s background, concomitant drug use, and drug type (e.g., opioids and antidementia drugs). We hope that the causal association of anticholinergic AEs and drugs can be clarified by performing research studies combined with several methods of analysis.

## Supporting information

S1 Table List of drugs whose −log *P* is 308 in the FAERS ALL result. Drugs whose −log *P* is 308 and lnRORs were greater than 0 in the FAERS ALL result. These drugs are listed in descending order of ROR.
(DOCX)

S2 Table List of drugs whose −log *P* is 308 in the FAERS CNS result. Drugs whose −log *P* is 308 and lnRORs were greater than 0 in the FAERS CNS result. These drugs are listed in descending order of ROR.
(DOCX)

S3 Table List of drugs whose −log *P* is 308 in the FAERS PNS result. Drugs whose −log *P* is 308 and lnRORs were greater than 0 in the FAERS PNS result. These drugs are listed in descending order of ROR.
(DOCX)

## References

[1] Hajjar ER, Hersh LR, Gray SL. Chapter e23: Prescribing in the Older Adult. In: Joseph DiPiro, Gary, Michael Posey L., Stuart T. Haines, Thomas D. Nolin, Vicki Ellingrod, editors. Pharmacotherapy: a pathophysiologic approach. Eleventh edition. New York: McGraw-Hill Medical; 2020. p. 69.

[2] Donohoe KL, Price ET, Gendron TL, Slattum PW. Chapter e22: Geriatrics: The Aging Process in Humans and Its Effects on Physiology, In: Joseph DiPiro, Gary Yee, Posey L. Michael, Stuart T. Haines, Thomas D. Nolin, Vicki El-lingrod, editors. Pharmacotherapy: a pathophysiologic approach. Eleventh edition. New York: McGraw-Hill Medical; 2020. p. 67.

[3] Laatikainen O, Sneck S, Bloigu R, Lahtinen M, Lauri T, Turpeinen M. Hospitalizations Due to Adverse Drug Events in the Elderly-A Retrospective Register Study. Front Pharmacol. 2016;7:358. doi: 10.3389/fphar.2016.00358 27761112 PMC5051318

[4] Mangoni AA, Jackson SH. Age-related changes in pharmacokinetics and pharmaco-dynamics: basic principles and practical applications. Br J Clin Pharmacol. 2004;57(1):6–14. doi: 10.1046/j.1365-2125.2003.02007.x 14678335 PMC1884408

[5] Villalba-Moreno AM, Alfaro-Lara ER, Pérez-Guerrero MC, Nieto-Martín MD, San-tos-Ramos B. Systematic review on the use of anticholinergic scales in polypathological patients [published correction appears in Arch Gerontol Geriatr. 2016 May-Jun;64:178–80]. Arch Gerontol Geriatr. 2016;62:1–8. doi: 10.1016/j.archger.2015.10.002 26518612

[6] Feinberg M. The problems of anticholinergic adverse effects in older patients. Drugs Aging. 1993;3(4):335–348. doi: 10.2165/00002512-199303040-00004 8369593

[7] Kersten H, Wyller TB. Anticholinergic drug burden in older people’s brain—how well is it measured?. Basic Clin Pharmacol Toxicol. 2014;114(2):151–159. doi: 10.1111/bcpt.12140 24112192

[8] Kouladjian L, Gnjidic D, Chen TF, Mangoni AA, Hilmer SN. Drug Burden Index in older adults: theoretical and practical issues. Clin Interv Aging. 2014; 9:1503–1515. doi: 10.2147/CIA.S66660 25246778 PMC4166346

[9] Wawruch M, Macugova A, Kostkova L, Luha J, Dukat A, Murin J, et al. The use of medications with anticholinergic properties and risk factors for their use in hospitalised elderly patients. Pharmacoepidemiol Drug Saf. 2012;21(2):170–176. doi: 10.1002/pds.2169 21671440

[10] Collamati A, Martone AM, Poscia A, Brandi V, Celi M, Marzetti E, et al. Anticholinergic drugs and negative outcomes in the older population: from biological plausibility to clinical evidence. Aging Clin Exp Res. 2016;28(1):25–35. doi: 10.1007/s40520-015-0359-7 25930085

[11] Wilson NM, Hilmer SN, March LM, et al. Associations between drug burden index and falls in older people in residential aged care. J Am Geriatr Soc. 2011;59(5):875–880. doi: 10.1111/j.1532-5415.2011.03386.x 21539525

[12] Fox C, Richardson K, Maidment ID, Savva GM, Matthews FE, Smithard D, et al. Anticholinergic medication use and cognitive impairment in the older population: the medical research council cognitive function and ageing study. J Am Geriatr Soc. 2011;59(8):1477–1483. doi: 10.1111/j.1532-5415.2011.03491.x 21707557

[13] Durán CE, Azermai M, Vander Stichele RH. Systematic review of anticholinergic risk scales in older adults. Eur J Clin Pharmacol. 2013;69(7):1485–1496. doi: 10.1007/s00228-013-1499-3 23529548

[14] Lisibach A, Benelli V, Ceppi MG, Waldner-Knogler K, Csajka C, Lutters M. Quality of anticholinergic burden scales and their impact on clinical outcomes: a systematic review. Eur J Clin Pharmacol. 2021;77(2):147–162. doi: 10.1007/s00228-020-02994-x 33011824 PMC7803697

[15] Ancelin ML, Artero S, Portet F, Dupuy AM, Touchon J, Ritchie K. Non-degenerative mild cognitive impairment in elderly people and use of anticholinergic drugs: longitudinal cohort study. BMJ. 2006;332(7539):455–459. doi: 10.1136/bmj.38740.439664.DE 16452102 PMC1382539

[16] Boustani M, Campbell N, Munger S, Maidment I, Fox C. Impact of anticholinergics on the aging brain: a review and practical application. Aging Health. 2008;4(3):311–320.

[17] Rudolph JL, Salow MJ, Angelini MC, McGlinchey RE. The anticholinergic risk scale and anticholinergic adverse effects in older persons. Arch Intern Med. 2008;168(5):508–513. doi: 10.1001/archinternmed.2007.106 18332297

[18] Lounkine E, Keiser MJ, Whitebread S, Mikhailov D, Hamon J, Jenkins JL, et al. Large-scale prediction and testing of drug activity on side-effect targets. Nature. 2012;486(7403):361–367. doi: 10.1038/nature11159 22722194 PMC3383642

[19] Kaneko S, Nagashima T. Drug repositioning and target finding based on clinical evidence. Biol Pharm Bull. 2020;43(3):362–365. doi: 10.1248/bpb.b19-00929 32115497

[20] Taylor-Rowan M, Edwards S, Noel-Storr AH, McCleery J, Myint PK, Soiza R, et al. Anticholinergic burden (prognostic factor) for prediction of dementia or cognitive decline in older adults with no known cognitive syndrome. Cochrane Database Syst Rev. 2021;5(5):CD013540. doi: 10.1002/14651858.CD013540.pub2 34097766 PMC8169439

[21] Nakham A, Myint PK, Bond CM, Newlands R, Loke YK, Cruickshank M. Interventions to reduce anticholinergic burden in adults aged 65 and older: a systematic review. J Am Med Dir Assoc. 2020;21(2):172–180. doi: 10.1016/j.jamda.2019.06.001 31351858

[22] NCBI [Internet]. MeSH database: adverse drug reaction reporting systems. [cited 2020 Nov 24]. Available from: https://www.ncbi.nlm.nih.gov/mesh/?term = Adverse+Drug+Reaction+Reporting+Systems.

[23] van Puijenbroek EP, van Grootheest K, Diemont WL, Leufkens HG, Egberts AC. Determinants of signal selection in a spontaneous reporting system for adverse drug reactions. Br J Clin Pharmacol. 2001;52(5):579–586. doi: 10.1046/j.0306-5251.2001.01501.x 11736867 PMC2014608

[24] Almenoff JS, Pattishall EN, Gibbs TG, DuMouchel W, Evans SJ, Yuen N. Novel statistical tools for monitoring the safety of marketed drugs. Clin Pharmacol Ther. 2007;82(2):157–166. doi: 10.1038/sj.clpt.6100258 17538548

[25] By the 2019 American Geriatrics Society Beers Criteria^®^ Update Expert Panel. American Geriatrics Society 2019 updated AGS Beers Criteria^®^ for potentially inappropriate medication use in older adults. J Am Geriatr Soc. 2019;67(4):674–694. doi: 10.1111/jgs.15767 30693946

[26] Food and Drug Administration (FDA) [Internet]. Questions and answers on FDA’s Adverse Event Reporting System (FAERS). [cited 2020 Nov 24]. Available from: https://www.fda.gov/drugs/surveillance/questions-and-answers-fdas-adverse-event-reporting-system-faers.

[27] Pharmaceuticals and Medical Devices Agency [Internet]. Information about the case report that an adverse reaction is suspected. [cited 2021 Mar 19]. Available from: https://www.pmda.go.jp/safety/info-services/drugs/adr-info/suspected-adr/0005.html. Japanese.

[28] Nomura K, Takahashi K, Hinomura Y, Kawaguchi G, Matsushita Y, Marui H, et al. Effect of database profile variation on drug safety assessment: an analysis of spontaneous adverse event reports of Japanese cases. Drug Des Devel Ther. 2015;9:3031–3041. doi: 10.2147/DDDT.S81998 26109846 PMC4472069

[29] MedDRA Maintenance and Support Services Organization (MSSO) [Internet]. Medical Dictionary for Regulatory Activities (MedDRA). [cited 2021 Feb 4]. Available from: https://www.meddra.org.

[30] U.S. Food and Drug Administration (FDA) Center for Drug Evaluation and Research (CDER) Office of Surveillance and Epidemiology (OSE). “ASC_NTS.DOC” file for the quarterly data extract (QDE) from the FDA Adverse Event Reporting System (FAERS); last revised June 2016.

[31] Pharmaceuticals and Medical Devices Agency [Internet]. Information provided on the dataset download page. [cited 2021 Mar 19]. Available from: https://www.pmda.go.jp/safety/info-services/drugs/adr-info/suspected-adr/0004.html. Japanese.

[32] Food and Drug Administration (FDA) [Internet]. Drugs@FDA data files. [cited 2021 Feb 4]. Available from: https://www.fda.gov/drugs/drug-approvals-and-databases/drugsfda-data-files.

[33] DrugBank [Internet]. DrugBank release version 5.1.8. [cited 2021 Feb 4]. Available from: https://go.drugbank.com/.

[34] Wishart DS, Feunang YD, Guo AC, Lo EJ, Marcu A, Grant JR, et al. DrugBank 5.0: a major update to the DrugBank database for 2018. Nucleic Acids Res. 2017 Nov 8. 10.1093/nar/gkx1037 PMC5753335 29126136

[35] Hirooka T, Yamada M. Evaluation of AEs risk using the “Japanese Adverse Drug Event Report database” of PMDA. SAS User General Assembly 2012 Proceedings. 2012: 263–270. Japanese.

[36] Nagai J, Uesawa Y, Shimamura R, Kagaya H. Characterization of the adverse effects induced by acetaminophen and nonsteroidal anti-inflammatory drugs based on the analysis of the Japanese Adverse Drug Event Report Database. Clin J Pain. 2017;33(8):667–675. doi: 10.1097/AJP.0000000000000457 27898459 PMC5497783

[37] van Puijenbroek EP, Bate A, Leufkens HG, Lindquist M, Orre R, Egberts AC. A comparison of measures of disproportionality for signal detection in spontaneous reporting systems for adverse drug reactions. Pharmacoepidemiol Drug Saf. 2002; 11:3–10. doi: 10.1002/pds.668 11998548

[38] Watanabe H, Matsushita Y, Watanabe A, Maeda T, Nukui K, Ogawa Y, et al. Early detection of important safety information—recent methods for signal detection. Jpn J Biomet. 2004; 25:37–60. Japanese.

[39] Yu Y, Chen J, Li D, Wang L, Wang W, Liu H. Systematic analysis of adverse event reports for sex differences in adverse drug events. Sci Rep. 2016; 6:24955. doi: 10.1038/srep24955 27102014 PMC4840306

[40] SAS Institute Inc. Chapter 3 Distributions. JMP^®^ 14 Basic Analysis. Cary, NC: SAS Institute Inc.; 2018. p. 58–59.

[41] Matsuda S, Aoki K, Kawamata T, Kimotsuki T, Kobayashi T, Kuriki H, et al. Bias in spontaneous reporting of adverse drug reactions in Japan. PLoS One. 2015;10: e0126413. doi: 10.1371/journal.pone.0126413 25933226 PMC4416713

[42] Carnahan RM, Lund BC, Perry PJ, Pollock BG, Culp KR. The Anticholinergic Drug Scale as a measure of drug-related anticholinergic burden: associations with serum anticholinergic activity. J Clin Pharmacol. 2006;46(12):1481–1486. doi: 10.1177/0091270006292126 17101747

[43] Han L, Agostini JV, Allore HG. Cumulative anticholinergic exposure is associated with poor memory and executive function in older men. J Am Geriatr Soc. 2008;56(12):2203–2210. doi: 10.1111/j.1532-5415.2008.02009.x 19093918 PMC3952110

[44] Chew ML, Mulsant BH, Pollock BG, Lehman ME, Greenspan A, Mahmoud RA, Kirshner MA, Sorisio DA, Bies RR, Gharabawi G. Anticholinergic activity of 107 medications commonly used by older adults. J Am Geriatr Soc. 2008;56(7):1333–1341. doi: 10.1111/j.1532-5415.2008.01737.x 18510583

[45] Ehrt U, Broich K, Larsen JP, Ballard C, Aarsland D. Use of drugs with anticholinergic effect and impact on cognition in Parkinson’s disease: a cohort study. J Neurol Neurosurg Psychiatry. 2010;81(2):160–165. doi: 10.1136/jnnp.2009.186239 19770163

[46] Sittironnarit G, Ames D, Bush AI, Faux N, Flicker L, Foster J, Hilmer S, Lautenschlager NT, Maruff P, Masters CL, Martins RN, Rowe C, Szoeke C, Ellis KA; AIBL research group. Effects of anticholinergic drugs on cognitive function in older Australians: results from the AIBL study. Dement Geriatr Cogn Disord. 2011;31(3):173–178. doi: 10.1159/000325171 21389718

[47] Miyachi T, Ozaki A, Saito H, Sawano T, Tanimoto T, Crump A. Opioids: A ‘crisis’ of too much or not enough—or simply how rich you are and where you live? Eur J Pain. 2021;25(6):1181–1194. doi: 10.1002/ejp.1767 33822443

[48] Lozano-Ortega G, Szabo SM, Cheung A, Suehs B, Caplan EO, Wagg A, et al. An evaluation of longitudinal measures of anticholinergic exposure for application in retrospective administrative data analyses. Adv Ther. 2019;36(9):2247–2259. doi: 10.1007/s12325-019-01035-z 31385284 PMC6822845

[49] Kersten H, Molden E, Willumsen T, Engedal K, Bruun Wyller T. Higher anticholinergic drug scale (ADS) scores are associated with peripheral but not cognitive markers of cholinergic blockade. Cross sectional data from 21 Norwegian nursing homes [published correction appears in Br J Clin Pharmacol. 2013 Apr;75(4):1171–2]. Br J Clin Pharmacol. 2013;75(3):842–849. 10.1111/j.1365-2125.2012.04411.x PMC3575951 22924454

[50] Fox C, Livingston G, Maidment ID, Coulton S, Smithard DG, Boustani M, et al. The impact of anticholinergic burden in Alzheimer’s dementia-the LASER-AD study. Age Ageing. 2011;40(6):730–735. doi: 10.1093/ageing/afr102 21926432

[51] The Japan Geriatrics Society [Internet]. List of drugs to be prescribed with special caution (version February 2017); [cited 2021 Sep 11]. Available from: https://www.jpn-geriat-soc.or.jp/info/topics/20150427_01.html. Japanese.

[52] Jun K, Hwang S, Ah YM, Suh Y, Lee JY. Development of an anticholinergic burden scale specific for Korean older adults. Geriatr Gerontol Int. 2019;19(7):628–634. doi: 10.1111/ggi.13680 31033150

[53] Pharmaceuticals and Medical Devices Agency. Guidelines on the implementation of drug epidemiological studies in the safety evaluation of drugs using a database or the like of pharmaceutical information. Chiyoda-ku, Tokyo, Japan: Pharmaceuticals and Medical Devices Agency; 2014:6–7. Japanese.

[54] Michel C, Scosyrev E, Petrin M, Schmouder R. Can disproportionality analysis of post-marketing case reports be used for comparison of drug safety profiles? Clin Drug Investig. 2017;37(5):415–422. doi: 10.1007/s40261-017-0503-6 28224371

[55] Astellas Pharma Global Development, Inc. [Internet]. Phase III Study of YM178: a double-blind group comparison study in patients with overactive bladder; c2013- 2018 [cited 2021 Jul 7]. Available from: https://astellasclinicalstudyresults.com/hcp/study.aspx?ID=193.

