# Supplementary material for: Analysis of anticholinergic adverse effects using two large databases: The US Food and Drug Administration Adverse Event Reporting System database and the Japanese Adverse Drug Event Report database
Source: PLoS One. 2021 Dec 2;16(12):e0260980. doi: 10.1371/journal.pone.0260980 (PMC8638968; doi:10.1371/journal.pone.0260980)
Supplement: S1 Table — Drugs whose −log P is 308 and lnRORs were greater than 0 in the FAERS ALL result. These drugs are listed in descending order of ROR. (DOCX) [file pone.0260980.s001.docx]

S1 Table.

|  | drug | a^a)^ | (a/(a+c^b)^)) ×100 | ROR^c)^ | 95% CI^d)^ |
| --- | --- | --- | --- | --- | --- |
| 1 | darifenacin | 965 | 0.09 | 16.3 | (15.2- 17.5) |
| 2 | sevelamer | 2,727 | 0.25 | 15.9 | (15.2- 16.5) |
| 3 | trospium | 466 | 0.04 | 14.6 | (13.3- 16.1) |
| 4 | solifenacin | 4,913 | 0.44 | 12.3 | (12.0- 12.7) |
| 5 | fesoterodine | 2,713 | 0.24 | 12.1 | (11.7- 12.6) |
| 6 | patiromer | 3,197 | 0.29 | 8.1 | (7.8- 8.4) |
| 7 | tolterodine | 1,778 | 0.16 | 7.1 | (6.8- 7.5) |
| 8 | memantine | 2,332 | 0.21 | 6.2 | (5.9- 6.4) |
| 9 | mirabegron | 2,736 | 0.25 | 5.2 | (5.0- 5.4) |
| 10 | pimavanserin | 6,080 | 0.55 | 4.8 | (4.7- 5.0) |
| 11 | telotristat ethyl | 1,030 | 0.09 | 4.5 | (4.2- 4.8) |
| 12 | oxybutynin | 1,411 | 0.13 | 4.5 | (4.2- 4.7) |
| 13 | erenumab | 3,323 | 0.30 | 4.2 | (4.1- 4.3) |
| 14 | niraparib | 7,241 | 0.65 | 4.2 | (4.1- 4.3) |
| 15 | orlistat | 2,346 | 0.21 | 3.2 | (3.1- 3.3) |
| 16 | finasteride | 3,868 | 0.35 | 3.1 | (3.0- 3.1) |
| 17 | amitriptyline | 2,292 | 0.21 | 3.0 | (2.9- 3.2) |
| 18 | haloperidol | 2,706 | 0.24 | 2.9 | (2.8- 3.0) |
| 19 | mirtazapine | 3,422 | 0.31 | 2.8 | (2.7- 2.9) |
| 20 | lorazepam | 3,509 | 0.32 | 2.7 | (2.6- 2.8) |
| 21 | carbidopa | 8,265 | 0.74 | 2.5 | (2.4- 2.6) |
| 22 | levodopa | 8,488 | 0.76 | 2.4 | (2.4- 2.5) |
| 23 | clozapine | 9,497 | 0.86 | 2.4 | (2.4- 2.5) |
| 24 | olanzapine | 8,252 | 0.74 | 2.4 | (2.3- 2.4) |
| 25 | duloxetine | 11,375 | 1.02 | 2.4 | (2.3- 2.4) |
| 26 | tiotropium | 6,129 | 0.55 | 2.3 | (2.3- 2.4) |
| 27 | zolpidem | 2,901 | 0.26 | 2.3 | (2.2- 2.4) |
| 28 | tramadol | 5,054 | 0.46 | 2.3 | (2.2- 2.4) |
| 29 | olmesartan | 4,287 | 0.39 | 2.1 | (2.1- 2.2) |
| 30 | enzalutamide | 4,762 | 0.43 | 2.1 | (2.0- 2.2) |
| 31 | bupropion | 3,907 | 0.35 | 2.1 | (2.0- 2.1) |
| 32 | citalopram | 3,719 | 0.34 | 2.0 | (2.0- 2.1) |
| 33 | morphine | 5,678 | 0.51 | 1.9 | (1.9- 2.0) |
| 34 | venlafaxine | 4,914 | 0.44 | 1.8 | (1.8- 1.9) |
| 35 | quetiapine | 10,858 | 0.98 | 1.8 | (1.7- 1.8) |
| 36 | sertraline | 5,855 | 0.53 | 1.7 | (1.7- 1.8) |
| 37 | gabapentin | 7,038 | 0.63 | 1.7 | (1.7- 1.8) |
| 38 | oxycodone | 9,061 | 0.82 | 1.7 | (1.7- 1.7) |
| 39 | dimethyl fumarate | 7,703 | 0.69 | 1.6 | (1.6- 1.7) |
| 40 | pregabalin | 14,696 | 1.32 | 1.6 | (1.6- 1.6) |
| 41 | lenalidomide | 14,141 | 1.27 | 1.5 | (1.5- 1.6) |
| 42 | natalizumab | 10,613 | 0.96 | 1.5 | (1.5- 1.5) |

^a)^ It is the “a” in the two-by-two table (i.e., number of cases reporting the suspected AE and the suspected drug). ^b)^ It is the “c” in the two-by-two table (i.e., number of cases reporting with the suspected AE and without the suspected drug). ^c)^ Reporting odds ratio. ^d)^ 95% Confidence Interval.
