# Supplementary material for: Analysis of anticholinergic adverse effects using two large databases: The US Food and Drug Administration Adverse Event Reporting System database and the Japanese Adverse Drug Event Report database
Source: PLoS One. 2021 Dec 2;16(12):e0260980. doi: 10.1371/journal.pone.0260980 (PMC8638968; doi:10.1371/journal.pone.0260980)
Supplement: S2 Table — Drugs whose −log P is 308 and lnRORs were greater than 0 in the FAERS CNS result. These drugs are listed in descending order of ROR. (DOCX) [file pone.0260980.s002.docx]

S2 Table.

|  | drug | a^a)^ | (a/(a+c^b)^)) ×100 | ROR^c)^ | 95% CI^d)^ |
| --- | --- | --- | --- | --- | --- |
| 1 | memantine | 1,776 | 0.45 | 13.2 | (12.6- 13.9) |
| 2 | pimavanserin | 4,587 | 1.17 | 10.4 | (10.1- 10.7) |
| 3 | donepezil | 1,055 | 0.27 | 7.5 | (7.0- 8.0) |
| 4 | finasteride | 3,078 | 0.79 | 6.9 | (6.7- 7.2) |
| 5 | rivastigmine | 1,206 | 0.31 | 6.2 | (5.9- 6.6) |
| 6 | oseltamivir | 913 | 0.23 | 4.9 | (4.6- 5.2) |
| 7 | lorazepam | 2,120 | 0.54 | 4.6 | (4.4- 4.8) |
| 8 | zolpidem | 2,000 | 0.51 | 4.5 | (4.3- 4.7) |
| 9 | carbidopa | 5,257 | 1.34 | 4.5 | (4.4- 4.7) |
| 10 | lithium carbonate | 1,186 | 0.30 | 4.5 | (4.2- 4.8) |
| 11 | levodopa | 5,373 | 1.37 | 4.4 | (4.3- 4.5) |
| 12 | haloperidol | 1,428 | 0.37 | 4.3 | (4.1- 4.5) |
| 13 | mirtazapine | 1,679 | 0.43 | 3.8 | (3.7- 4.0) |
| 14 | duloxetine | 6,382 | 1.63 | 3.8 | (3.7- 3.9) |
| 15 | olanzapine | 4,357 | 1.11 | 3.6 | (3.5- 3.7) |
| 16 | tramadol | 2,576 | 0.66 | 3.3 | (3.2- 3.5) |
| 17 | natalizumab | 7,966 | 2.04 | 3.2 | (3.2- 3.3) |
| 18 | topiramate | 1,701 | 0.43 | 3.2 | (3.0- 3.4) |
| 19 | valproic acid | 2,450 | 0.63 | 3.1 | (3.0- 3.3) |
| 20 | levetiracetam | 2,160 | 0.55 | 2.7 | (2.6- 2.8) |
| 21 | gabapentin | 3,761 | 0.96 | 2.6 | (2.5- 2.7) |
| 22 | paroxetine | 2,843 | 0.73 | 2.6 | (2.5- 2.7) |
| 23 | sertraline | 2,952 | 0.75 | 2.5 | (2.4- 2.6) |
| 24 | morphine | 2,567 | 0.66 | 2.4 | (2.4- 2.5) |
| 25 | dimethyl fumarate | 3,945 | 1.01 | 2.4 | (2.3- 2.4) |
| 26 | clozapine | 3,128 | 0.80 | 2.2 | (2.2- 2.3) |
| 27 | quetiapine | 4,702 | 1.20 | 2.2 | (2.1- 2.2) |
| 28 | pregabalin | 6,553 | 1.68 | 2.1 | (2.0- 2.1) |
| 29 | interferon beta-1a | 6,696 | 1.71 | 2.0 | (1.9- 2.0) |

^a)^ It is the “a” in the two-by-two table (i.e., number of cases reporting the suspected AE and the suspected drug). ^b)^ It is the “c” in the two-by-two table (i.e., number of cases reporting with the suspected AE and without the suspected drug). ^c)^ Reporting odds ratio. ^d)^ 95% Confidence Interval.
