# Supplementary material for: Analysis of anticholinergic adverse effects using two large databases: The US Food and Drug Administration Adverse Event Reporting System database and the Japanese Adverse Drug Event Report database
Source: PLoS One. 2021 Dec 2;16(12):e0260980. doi: 10.1371/journal.pone.0260980 (PMC8638968; doi:10.1371/journal.pone.0260980)
Supplement: S3 Table — Drugs whose −log P is 308 and lnRORs were greater than 0 in the FAERS PNS result. These drugs are listed in descending order of ROR. (DOCX) [file pone.0260980.s003.docx]

S3 Table.

|  | drug | a^a)^ | (a/(a+c^b)^)) ×100 | ROR^c)^ | 95% CI^d)^ |
| --- | --- | --- | --- | --- | --- |
| 1 | sevelamer | 2701 | 0.38 | 24.3 | (23.4- 25.3) |
| 2 | darifenacin | 922 | 0.13 | 24.0 | (22.3- 25.7) |
| 3 | trospium | 391 | 0.05 | 18.6 | (16.7- 20.6) |
| 4 | solifenacin | 4473 | 0.62 | 17.2 | (16.7- 17.8) |
| 5 | fesoterodine | 2500 | 0.35 | 17.2 | (16.5- 17.9) |
| 6 | patiromer | 3149 | 0.44 | 12.3 | (11.9- 12.8) |
| 7 | tolterodine | 1575 | 0.22 | 9.7 | (9.2- 10.2) |
| 8 | mirabegron | 2475 | 0.34 | 7.2 | (6.9- 7.5) |
| 9 | telotristat ethyl | 1001 | 0.14 | 6.8 | (6.4- 7.2) |
| 10 | erenumab | 3142 | 0.44 | 6.1 | (5.9- 6.4) |
| 11 | niraparib | 6704 | 0.93 | 6.0 | (5.8- 6.1) |
| 12 | oxybutynin | 1108 | 0.15 | 5.4 | (5.1- 5.7) |
| 13 | orlistat | 2286 | 0.32 | 4.8 | (4.6- 5.0) |
| 14 | tolvaptan | 1031 | 0.14 | 4.4 | (4.2- 4.7) |
| 15 | rucaparib | 1860 | 0.26 | 3.8 | (3.6- 4.0) |
| 16 | loperamide | 1750 | 0.24 | 3.5 | (3.3- 3.7) |
| 17 | tiotropium | 5574 | 0.78 | 3.3 | (3.2- 3.4) |
| 18 | olmesartan | 4076 | 0.57 | 3.1 | (3.0- 3.2) |
| 19 | cabozantinib | 1823 | 0.25 | 2.9 | (2.8- 3.0) |
| 20 | nintedanib | 1886 | 0.26 | 2.8 | (2.6- 2.9) |
| 21 | clozapine | 6369 | 0.89 | 2.5 | (2.4- 2.6) |
| 22 | bupropion | 2836 | 0.39 | 2.3 | (2.2- 2.4) |
| 23 | dupilumab | 2925 | 0.41 | 2.3 | (2.2- 2.4) |
| 24 | enzalutamide | 3303 | 0.46 | 2.2 | (2.2- 2.3) |
| 25 | palbociclib | 4183 | 0.58 | 2.1 | (2.0- 2.1) |
| 26 | levothyroxine | 4569 | 0.64 | 1.9 | (1.9- 2.0) |
| 27 | lenalidomide | 10539 | 1.47 | 1.8 | (1.7- 1.8) |

^a)^ It is the “a” in the two-by-two table (i.e., number of cases reporting the suspected AE and the suspected drug). ^b)^ It is the “c” in the two-by-two table (i.e., number of cases reporting with the suspected AE and without the suspected drug). ^c)^ Reporting odds ratio. ^d)^ 95% Confidence Interval.
